# Supplementary material for: Identification of Plitidepsin as Potent Inhibitor of SARS-CoV-2-Induced Cytopathic Effect After a Drug Repurposing Screen
Source: Front Pharmacol. 2021 Mar 25;12:646676. doi: 10.3389/fphar.2021.646676 (PMC8033486; doi:10.3389/fphar.2021.646676)
Supplement: Supplementary file 1 [file table1.pdf]

ACTIVITY	DRUG	IC_{50} / CC_{50} µM (Mean +/-SD)	Mode of Action	Previous Clinical Use	Vendor Origen
ENTRY	Hydroxychloroquine	9.3 +/- 11.1 / > 80	Clathrin-mediated endocitosys or pH-dependent viral fusion inhibitor	Malaria	Laboratorios Rubió
	Chloroquine	3.9 / > 25			Sigma Aldrich
	Amantadine	*Not calculated, but partialy active at 100* / > 100	Clathrin-mediated endocitosys inhibitor	Parkinson & influenza A	Sigma Aldrich
	Chlorpromazine (Largactil)	Not Active / > 18	Clathrin-mediated endocitosys inhibitor	Antipsychotic	Sanofi
	CA-074-Me	Not Active / > 34	Cathepsin inhibitor B	*Pre-Clinical*	Sigma Aldrich
	E-64d	*Not calculated, but partialy active at 100* / > 100	Cathepsin inhibitor B/L	*Pre-Clinical*	Sigma Aldrich
	Methyl-ß-cyclodextrin	*Not calculated, but active at 1000*	Cholesterol-removing agent, lipid raft disruption	*Not approved*	Sigma Aldrich
	NB-DNJ	*Not calculated, but active at 100* / > 100	Inhibits ceramide- glucosyltransferase and β-glucosidase 2	Gaucher disease & Juvenile Sandhoff disease	Calbiochem
	3’ Sialyllactose Na Salt	Not Active / 20 mM	Inhibits viral binding	*Pre-Clinical*	Carbosynth
	Niclosamide	Not Active / > 9	Beclin-1 stabilizer in autophagy	Helmints	Selleckchem
	Ciclesonide	Not Active / > 20	Glucocorticoid	Asthma	Selleckchem
	Arbidol HCl	Not Active / > 40	Fusion inhibitor?	Influenza	Selleckchem
	Tofacitinib (Xeljanx)	Not Active / > 100	JAK inhibitor	Rheumatoid arthritis	Pfizer
	Baricitinib	Not Active / > 85	JAK inhibitor	Rheumatoid arthritis	Selleckchem
	Camostat	Not Active / > 100	TMPRSS2 inhibitor	Chronic pancreatitis	Merck
	Alpha-1 Antitrypsin	*Not calculated, but active at 12.5 mg/ml* / > 12.5 mg/ml	Cellular protease inhibitor	Alpha-1 antitrypsin deficiency	Grifols

Supplementary Table 1
