# Supplementary material for: Identification of Plitidepsin as Potent Inhibitor of SARS-CoV-2-Induced Cytopathic Effect After a Drug Repurposing Screen
Source: Front Pharmacol. 2021 Mar 25;12:646676. doi: 10.3389/fphar.2021.646676 (PMC8033486; doi:10.3389/fphar.2021.646676)
Supplement: Supplementary file 2 [file table2.pdf]

DRUG 1	DRUG 2	DRUG 3	DRUG 4	Synergy	Toxicity in combination
Hydroxy-Cloroquine	Azithromycin			No	Similar
	Lopinavir			No	Similar
	Tipranavir			No	Similar
	Amprenavir			No	Similar
	Darunavir			No	Similar
	Baricitinib			No	Similar
	Tenofovir	Emtricitabine		No	Similar
	TAF	Emtricitabine		No	Similar
Remdesivir	Hydroxychloroquine			No	Similar
	Amantadine			No	Similar
	Chlorpromazine			No	Similar
	Baricitinib			No	Higher
	Tipranavir	Lopinavir		No	Similar
Lopinavir	Ritonavir			No	Higher
	Tipranavir			No	Higher
	Ritonavir	Tenofovir	Emtricitabine	No	Similar
	Ritonavir	Tenofovir Alafenamide	Emtricitabine	No	Similar
Tipranavir	Tenofovir	Emtricitabine		No	Similar
Tenofovir	Emtricitabine			No	Similar
Nelfinavir Mesylate Hydrate	Hydroxychloroquine			No	Similar
	Remdesivir			No	Similar
MDL28170	Hydroxychloroquine			No	Similar
	Remdesivir			No	Similar
	Nelfinavir Mesylate Hydrate			No	Similar
Plitidepsin	Hydroxychloroquine			No	Similar
	Remdesivir			No	Similar
	MDL 28170			No	Similar
	Nelfinavir Mesylate Hydrate			No	Similar

Supplementary Table 2
