# Supplementary material for: Identification of Plitidepsin as Potent Inhibitor of SARS-CoV-2-Induced Cytopathic Effect After a Drug Repurposing Screen
Source: Front Pharmacol. 2021 Mar 25;12:646676. doi: 10.3389/fphar.2021.646676 (PMC8033486; doi:10.3389/fphar.2021.646676)
Supplement: Supplementary file 3 [file table3.pdf]

ACTIVITY	DRUG	IC_{50} / CC_{50} µM (Mean +/-SD)	Mode of Action	Previous Clinical Use	Vendor Origen
POST-ENTRY	Remdesivir	2.2 +/-4.1 / > 85	RNA Polymerase inhibitor	Ebola Virus	Cayman Chemical
	Galdesivir	Not Active / 100	RNA Polymerase inhibitor	YFV	MedChemExpress
	Favipiravir	*Not calculated, but partially active at 100* / > 100	RNA polimerase inhibitor	Flavivirus, Arenavirus, Bunyavirus, Alphavirus	Quimigen
	Saquinavir	Not Active / > 28	Protease inhibitor	HIV-1	Reference standard HPLC
	Lopinavir	*Not calculated, but active at 20* / > 87	Protease inhibitor	HIV-1	Abbott
	Ritonavir	*Not active* / 20-100	Protease inhibitor	HIV-1	Abbott
	Tipranavir	*Not calculated, but active at 20* / > 70	Protease inhibitor	HIV-1	Reference standard HPLC
	Nelfinavir Mesylate Hydrate	*Not calculated, but active <10* / > 25	Protease inhibitor	HIV-1	Sigma Aldrich
	Amprenavir	*Not calculated, but active at 100* / > 100	Protease inhibitor	HIV-1	GSK
	Fosamprenavir Calcium	Not Active / > 25	Protease inhibitor	HIV-1	Sigma Aldrich
	Darunavir	*Not calculated, but partially active at 100* / > 100	Protease inhibitor	HIV-1	Sigma Aldrich
	Atazanavir Sulfate	Not Active / > 20	Protease inhibitor	HIV-1	Reference standard HPLC
	Tenofovir disoproxil fumarate	Not Active / > 100	Reverse Transcriptase inhibitor	HIV-1	Selleckchem
	Emtricitabine (Emtriva)	Not Active / > 100	Reverse Transcriptase inhibitor	HIV-1	Gilead
	Tenofovir Alafenamide	Not Active / > 100	Reverse Transcriptase inhibitor	HIV-1	Selleckchem
	Velpatasvir	Not Active / > 10	Iinhibitor of NS5A protein	HCV	Selleckchem
	Sofosbuvir	Not Active / > 100	Polymerase inhibitor	HCV	Selleckchem
	Boceprevir	Not Active / > 100	Protease inhibitor	HCV	Quimigen
	Vesatolimod	Not Active / > 20	TLR7 agonist	Hepatitis & HIV-1	MedChemExpress
	Interferon 2 alpha	8.1+/-0.7 $x10^2$ IU/mL / >100 $x10^2$ IU/mL	IFN stimulated antiviral proteins	Hepatitis & HIV-1	Sigma-Aldrich
	Interferon gamma	11.2 $x10^2$ IU/mL / >100 $x10^2$ IU/mL	IFN stimulated antiviral proteins	Granulomatous disease	Sigma-Aldrich
	Plitidepsin	0.06 +/- 0.02 / > 0.1	Targets eukaryotic Elongation Factor 1A2 (eEF1A2)	Multiple myeloma	PharmaMar

Supplementary Table 3
