# Supplementary material for: Identification of Plitidepsin as Potent Inhibitor of SARS-CoV-2-Induced Cytopathic Effect After a Drug Repurposing Screen
Source: Front Pharmacol. 2021 Mar 25;12:646676. doi: 10.3389/fphar.2021.646676 (PMC8033486; doi:10.3389/fphar.2021.646676)
Supplement: Supplementary file 4 [file table4.pdf]

Bioinformatic ANALYSIS	DRUG	IC_{50} / CC_{50} µM (Mean +/-SD)	Mode of Action	Previous Clinical Use	Vendor Origen
	Salbutamol	Not Active	Predicted SARS-Cov-2 Protease inhibitor	Asthma	Sigma Aldrich
	Diclondazolic acid (Lonidamine)	Not Active	Predicted SARS-Cov-2 Protease inhibitor	Anticancer	Abcam
	Thiocolchicoside	Not Active	Predicted SARS-Cov-2 Protease inhibitor	Skeletal muscle relaxant	Sigma Aldrich
	Morphothiadine	Not Active / 54	Predicted SARS-Cov-2 Protease inhibitor	Hepatitis B	Quimigen
	Ingliforib	Not Active	Predicted SARS-Cov-2 Protease inhibitor	Glycogen phosphorylase inhibitor	Quimigen
	Perampanel	Not Active	Predicted SARS-Cov-2 Protease inhibitor	Antiepileptic	Quimigen
	Montirelin trifluoroacetate salt	Not Active	Predicted SARS-Cov-2 Protease inhibitor	Thyrotropin-releasing hormone agonists	Sigma Aldrich
	Saquinavir	Not Active	Predicted SARS-Cov-2 Protease inhibitor	HIV-1	Reference standard HPLC
	Pamicogrel	Not Active / 6.4	Predicted SARS-Cov-2 Protease inhibitor	Antiplatelet aggregation	Quimigen
	Pomalidomide	Not Active	Predicted SARS-Cov-2 Protease inhibitor	Anticonvulsants	Sigma Aldrich
	Laflunimus Sodium Salt	Not Active	Predicted SARS-Cov-2 Protease inhibitor	Anti-inflammatory	Quimigen

Supplementary Table 4
