# Supplementary material for: Identification of Plitidepsin as Potent Inhibitor of SARS-CoV-2-Induced Cytopathic Effect After a Drug Repurposing Screen
Source: Front Pharmacol. 2021 Mar 25;12:646676. doi: 10.3389/fphar.2021.646676 (PMC8033486; doi:10.3389/fphar.2021.646676)
Supplement: Supplementary file 5 [file table5.pdf]

ACTIVITY	DRUG	IC_{50} / CC_{50} µM (Mean +/-SD)	Mode of Action	Previous Clinical Use	Vendor Origen
UNKNOWN	Azithromycin (Zitromax)	Not Active / > 100	Antibiotic	Bacteria	Pfizer
	Doxycycline (Anaclosil)	Not Active / > 100	Antibiotic	Bacteria	Reig
	Eravacycline (Xerava)	Not Active / > 4	Antibiotic	Resistent bacteria	Tetraphase Pharmaceuticals
	Quinacrine dihydrochloride	Not Active / > 6	Inhibitor of NF-kappaB	Parasites	Sigma Aldrich
	Ivermectin (Stromectol)	Not Active **/** > 2	Nuclear import inhibitor	Parasites	MSD
	Mefloquine hydrochloride	Not Active / > 100	Phospholipid bilayer?	Malaria	Sigma Aldrich
	N-Acetil cystein (Flumil)	Not Active / > 100	Synthesis of glutathione	Influenza	Zambon
	Itraconazole	79.37 **/** > 100	Inhibits OSBP, which produces the membrane-bound viral replication organelles	Fungus	Janssen
	Fluconazol	Not Active **/** > 100	Antibiotic	Fungus	Franesius Kabi
	Famotidine	Not Active**/** > 100	Histamine-2 receptor antagonist	Gastric	Normon
	Cetirizine dihydorcloride	Not Active**/** > 100	Histamine-H1 receptor antagonist	Antihistaminic	Sigma Aldrich
	Colchicine	Not Active / 0.63	Anti mytotic	Gout attacks	Merck
	Palbociclib	Not Active**/** 2,7	CDK4/6 inhibitor	Breast cancer	Selleckchem
	Ribociclib	Not Active / > 20	CDK4/6 inhibitor	Breast cancer	Selleckchem
	Abenaciclib	Not Active **/** > 1	CDK4/6 inhibitor	Breast cancer	Selleckchem
	Silibinin	Not active / > 20	?	Liver disease	Rottapharm Madaus
	Atorvastatin	Not active / > 20	HMG-CoA reductase inhibitor	Cardiovascular disease	Normon
	Fenofibrate	19.8 +/- 8 / > 100	Activates PPARα	Dyslipidemia	Lacer
	MDL 28170	0.14 +/- 0.06 / > 87	Calpain III inhibitor & Cathepsin B inhibitor	*Pre-Clinical*	Merck
	NPO-2142; -2143 & -2260	~ 0.54 / > 10	Calpain & Cathepsin inhibitors	*Pre-Clinical*	Landsteiner Genmed
	NPO-2138	*Not calculated, but partially active at 100* / > 10	Calpain & Cathepsin inhibitors	*Pre-Clinical*	Landsteiner Genmed

Supplementary Table 5
