# Supplementary figures and images for: Identification of Plitidepsin as Potent Inhibitor of SARS-CoV-2-Induced Cytopathic Effect After a Drug Repurposing Screen
Source: Front Pharmacol. 2021 Mar 25;12:646676. doi: 10.3389/fphar.2021.646676 (PMC8033486; doi:10.3389/fphar.2021.646676)

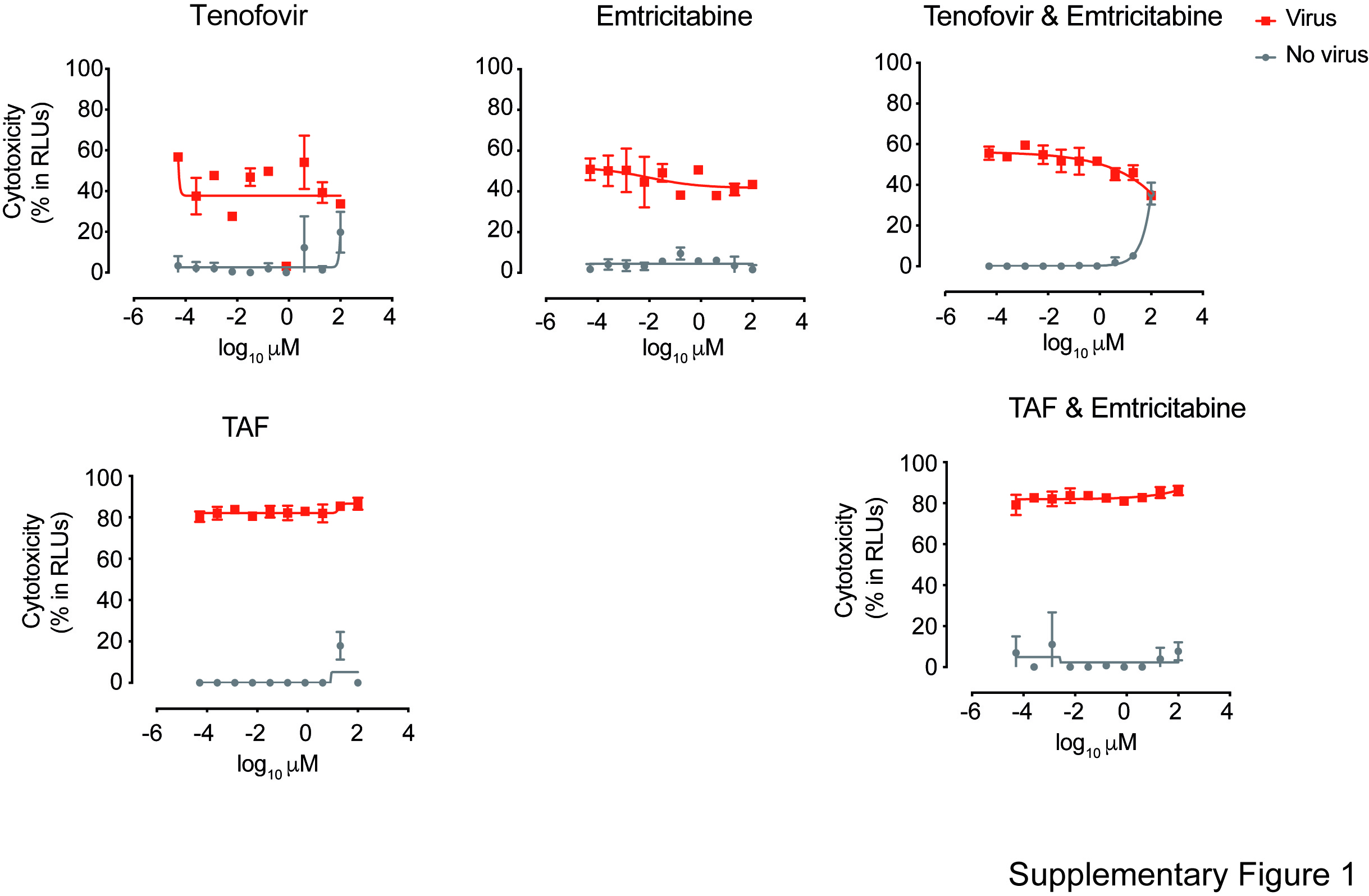

Supplement: Supplementary file 6 [file image1.jpeg]

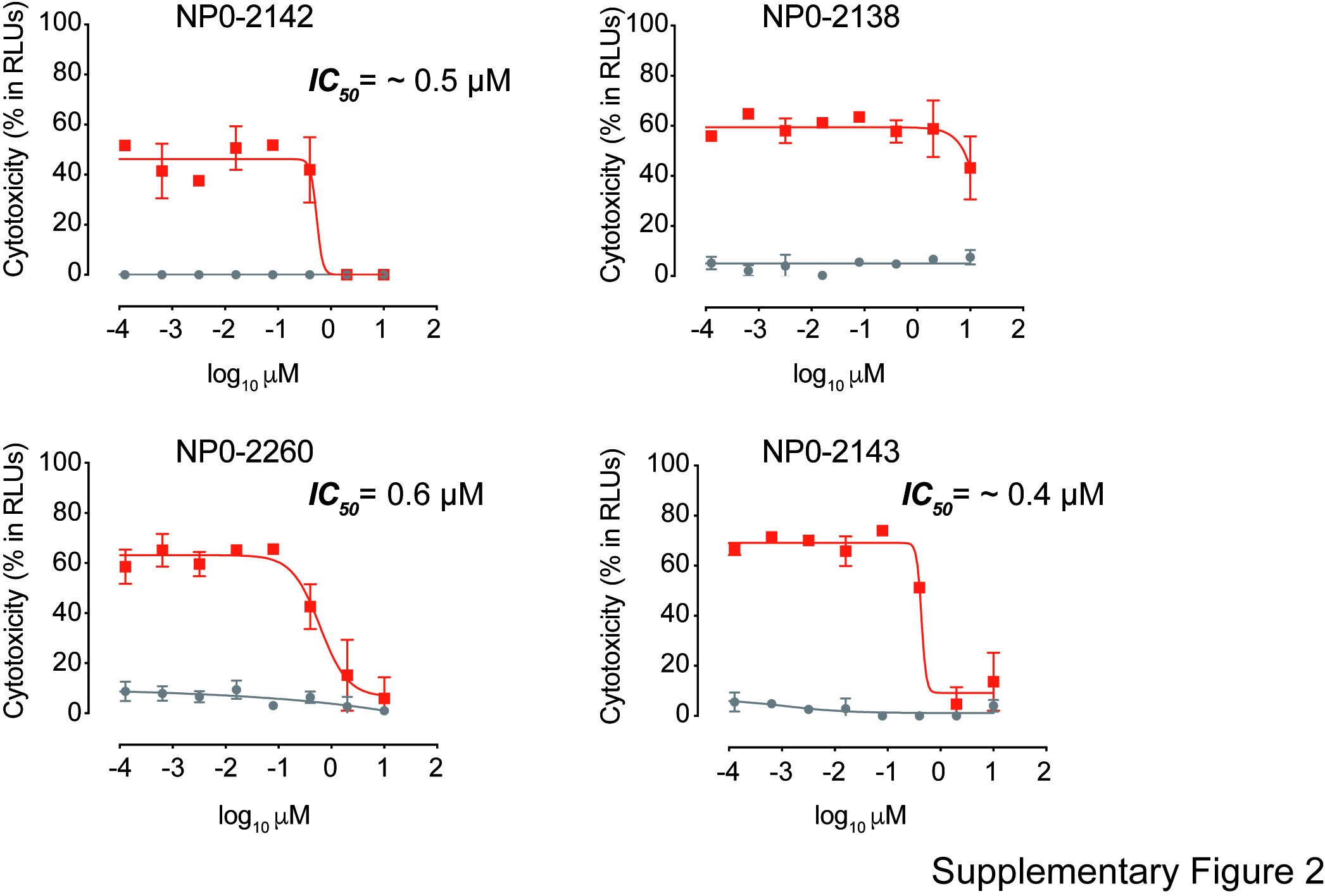

Supplement: Supplementary file 7 [file image2.jpeg]
